# Programmatic implementation of Ovicol ovitraps for* Aedes* spp. control in Colombia: operational adherence, effectiveness and comparisons with AGO and BG-Sentinel traps

**DOI:** 10.1186/s13071-026-07340-1

**Published:** 2026-03-13

**Authors:** Laureano Mosquera, Juan Osorio, Orley Herrera, Diego Montenegro

**Affiliations:** 1 Secretaria Departamental de Salud del Guaviare, 950007 San José del Guaviare, Colombia; 2 Secretaria Distrital de Salud de Santa Marta, 470001 Santa Marta, Colombia; 3 Grupo de Investigación, Salud y Tecnología 4.0. Fundación Chilloa, 470001 Santa Marta, Colombia; 4 Corporation Innovation Hub, 230001 Montería, Colombia; 5 Department of Microbiology and Environmental Toxicology, University of California at Santa Cruz, Santa Cruz, CA 95064 USA

**Keywords:** *Aedes aegypti*, *Aedes albopictus*, Ovitrap, Ovicidal control, Vector surveillance, Entomovirus

## Abstract

**Background:**

The global expansion of *Aedes aegypti* and *Aedes albopictus* mosquitoes has intensified arboviral epidemics, generating annual socio-economic losses exceeding 2 billion US dollars, mainly in the Americas. The desiccation-resistant egg stage plays a key role in mosquito persistence and spread, yet this developmental stage remains insufficiently targeted by current control strategies. In this context, the objective of this study was to evaluate the operational feasibility and effectiveness of a low-cost, handcrafted ovitrap (Ovicol) using two types of bioattractants, implemented by two territorial health entities (Entidades Territoriales de Salud [ETSs]) in Colombia, and to compare its performance with that of industrial autocidal gravid ovitraps (AGO) and BG-Sentinel (BGS) traps

**Methods:**

Ovicol traps were baited with bird seed or molasses + yeast bioattractants. In Santa Marta, traps were installed in public areas (markets, cemeteries, sports complexes) using diflubenzuron as insecticide; in San José del Guaviare, the traps were deployed in buildings (hotels, schools, health centers) using a combination of diflubenzuron and *Bacillus thuringiensis israelensis* (BTi) as insecticide treatment. Data were analyzed through descriptive statistics, analysis of variance/chi-square test, nonparametric tests and spatial cluster detection.

**Results:**

In Santa Marta, 41,677 eggs (100% *Ae. aegypti*) were eliminated and inactivated in 5 weeks. The Oviposition Positivity Index (OPI) ranged from 40.6% to 74.5%, with up to 88% of traps lost to the study at one site due to vandalism. In Guaviare, Ovicol traps registered oviposition within 24 h and outperformed AGO in terms of *Aedes* detection (65.2% vs. 30.8%, respectively; *r* = 0.87; *P* < 0.001). The fermented bird seed attractant achieved higher positivity (Kruskal–Wallis *H* = 9.42; *P* = 0.009). Compared with BGS traps, Ovicol showed superior stability in the field, with BGS traps limited by ≥ 70% rate of electrical disconnection during weekends, and low concordance (kappa: − 0.17).

**Conclusions:**

Ovicol is a cost-effective, operationally simple, and eco-sustainable tool for *Aedes* surveillance and control. By outperforming AGO and complementing BGS traps, Ovicol enhances spatiotemporal resolution for early microfocus detection and targeted response. Acting as a lethal trap, one diflubenzuron tablet (normally for 200 l) can treat approximately 800 Ovicol (0.25 L each), extending coverage to approximately 800 households and achieving an approximately 800-fold increase in larvicide efficiency. These findings support Ovicol’s incorporation into national programs to promote sustainable, community-driven integrated vector management.

**Graphical Abstract:**

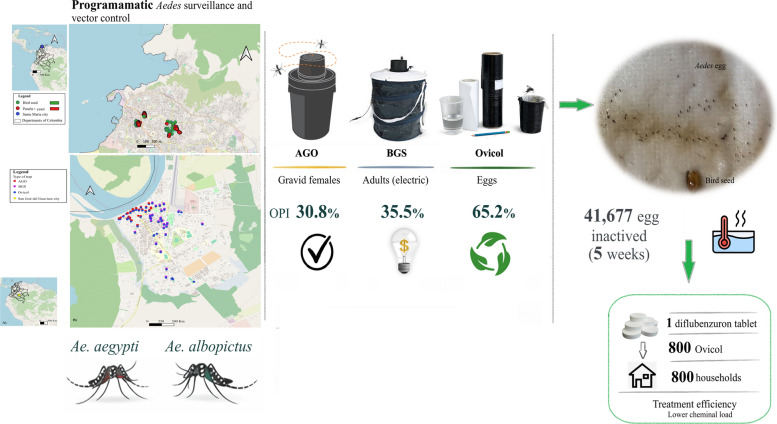

## Background

The genus *Aedes* includes more than 70 mosquito species [1], among which the African mosquito *Aedes aegypti* and the Asian mosquito *Aedes albopictus* have expanded far beyond their native ranges and gained notoriety for their remarkable ability to adapt to human-modified environments [2]. The global spread of *Ae. aegypti* has been linked to the slave trade during the colonial era [3], whereas the dissemination of *A. albopictus* has been largely driven by the transport of desiccation-resistant eggs in used tires [4]. Today, these species are established in nearly 170 countries across all continents except Antarctica [5].

The global economic burden associated with *Aedes*-borne diseases—primarily dengue, Zika, chikungunya and urban yellow fever—is estimated to exceed 94 billion US dollars (USD), with the Americas accounting for 47.4% of that cost [6]. In addition, both *Ae. aegypti* and *Ae. albopictus*species have developed resistance to more than 40 insecticidal compounds [7], making their control increasingly challenging.

*Aedes* mosquitoes have gained renewed relevance due to the ongoing yellow fever outbreaks reported in several countries of the Americas, particularly Bolivia, Brazil, Colombia and Peru, where a case fatality rate of 43.2% (83/192) was recorded between 2024 and early 2025 [8, 9]. Although most reported cases have originated from the sylvatic transmission cycle, involving other mosquito genera such as *Sabethes* and *Haemagogus* [8, 9], a latent risk of urbanization persists. This risk is well recognized, as *Ae. aegypti* and *Ae. albopictus* are competent vectors for the yellow fever virus in urbanized environments [10, 11], and the re-establishment of urban transmission cycles represents one of the most critical threats for public health systems in the region.

Among the current vector control strategies, sticky adult traps, such as the autocidal gravid ovitrap (AGO), have proven effective for capturing gravid *Aedes* females [12]. However, their epidemiological impact requires extensive coverage—typically exceeding 80% of households—to significantly reduce adult densities and transmission risk [13]. For sentinel surveillance and adult mosquito detection, the BG-Sentinel (BGS) trap has become a standard device that operates with electrical power and is designed to attract host-seeking females through visual and olfactory cues [14]. Despite their proven sensitivity, the large-scale use of these traps in routine vector surveillance and control programs (PRVCs) is constrained by cost, logistics and dependence on imported materials.

From a biological standpoint, the egg stage represents the least targeted phase of the *Aedes* life-cycle in integrated vector management (IVM) [15–17] despite their critical role in long-distance dispersal at continental scales [18, 19]. This is in part due to their resilience, as *Ae. aegypti* and *Ae. albopictus* eggs can remain viable for months under dry conditions [15, 20], and to the absence of effective ovicides and the limited persistence of larvicides relative to egg longevity.

To address these gaps, we recently demonstrated that targeting the egg stage through handmade ovitraps which combine water, bird seed (*Phalaris canariensis*) as a natural attractant and thermal control using hot water, offers a cost-effective (< USD 0.20 per unit), operationally simple and environmentally sustainable approach [21]. Building upon that experience, in the present study, we evaluated an optimized version of this trap, termed Ovicol, which has been designed to strengthen national surveillance programs through scalable, community-compatible implementation. In collaboration with local health authorities in Colombia, we assessed both the operational adherence and field performance of Ovicol under real-life programmatic conditions. Additionally, we tested a new bioattractant formulation, and the results were compared with those obtained using AGO and BGS traps, with the aim of enhancing the integration of Ovicol into national and subnational PRVC frameworks in arbovirus-endemic regions.

## Methods

### Sampling sites

A joint action plan was established and formalized with two territorial health entities (ETSs) in Colombia, namely the ETS of Santa Marta located in the Caribbean region and the ETS of San José del Guaviare in the Amazon region. Both regions are endemic areas for dengue and yellow fever [22, 23].

**Fig. 1:**
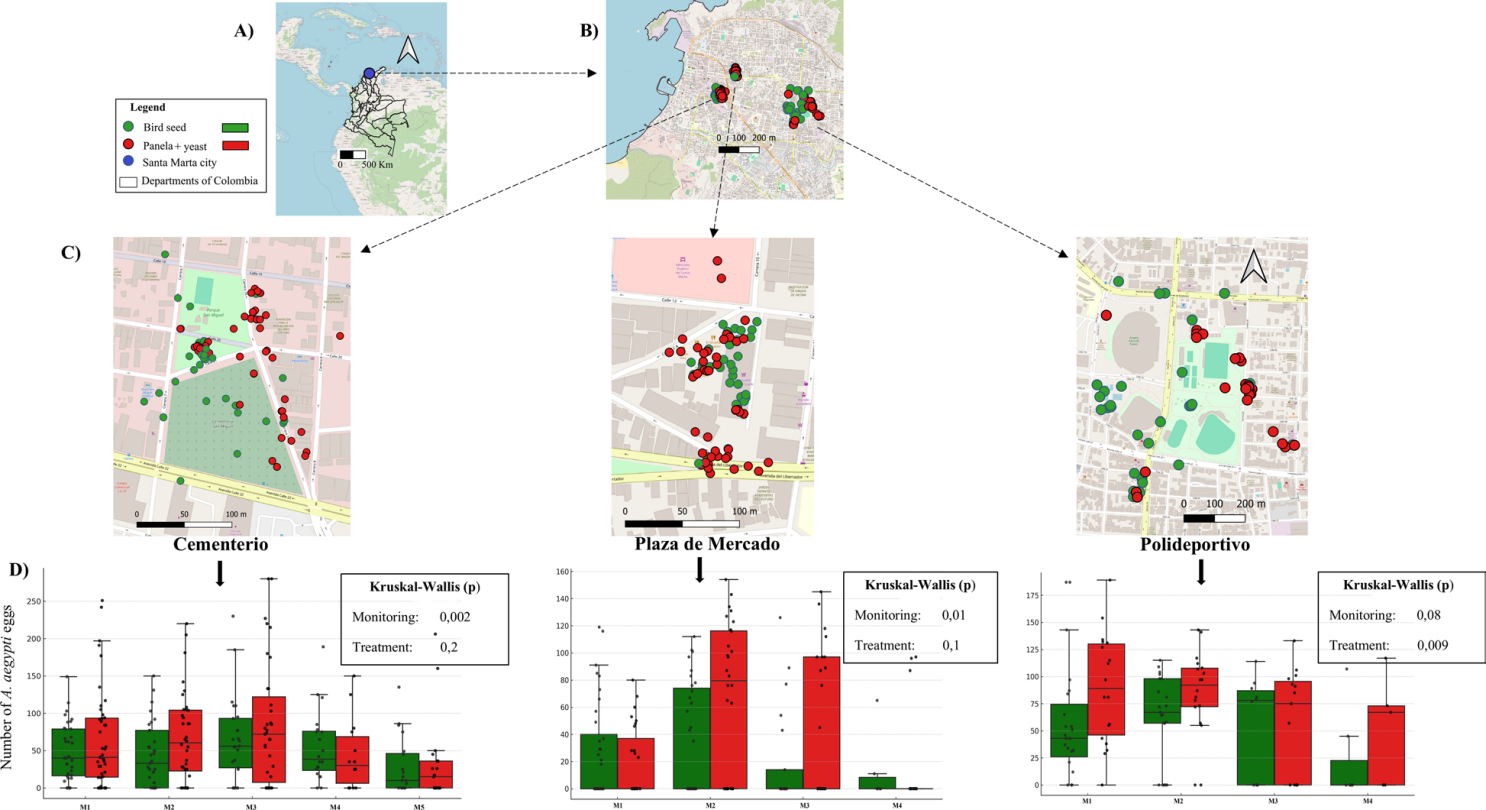
Temporal dynamics of *Aedes aegypti* oviposition in Ovicol traps using two types of bioattractants in public places/civic spaces of Santa Marta. **a** Location of Santa Marta in the Colombian Caribbean region. **b** Spatial distribution of Ovicol traps across the city of Santa Marta. **c** Geolocation of Ovicol traps according to bioattractant type (green and red filled circles). **d** Number of *Aedes* eggs captured by treatment type and monitoring week

In Santa Marta, Ovicol traps were deployed across five intensively used public/civic sites: Plaza de Mercado (the central marketplace), Cementerio San Miguel (San Miguel Cemetery), Parque del Agua (Water Park), Polideportivo (the municipal sports complex) and the peridomestic area of Villa Dania neighborhood. Ovitrap monitoring was conducted weekly from November to December 2024 by teams composed of one professional and two technicians (Fig. 1).

**Fig. 2:**
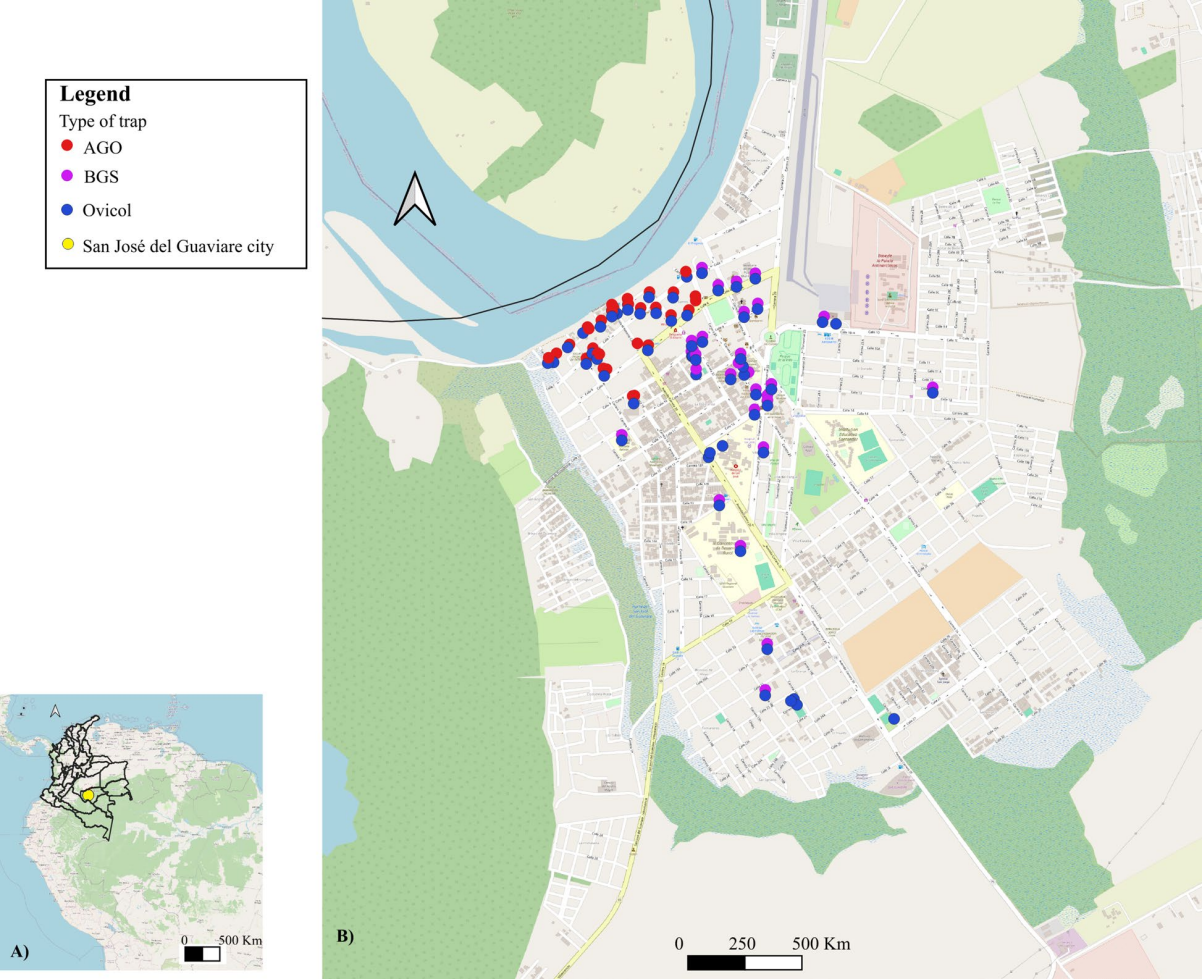
**a** Location of Guaviare in the Colombian Amazon. **b** Spatial distributions of Ovicol, BG-Sentinel and AGO traps across the city. AGO, Autocidal gravid ovitrap

In San José del Guaviare, two parallel study arms were conducted from March to April 2025: (i) Ovicol traps were tested on the premises or patios of two public facilities: Laboratorio de Salud Pública (Public Health Laboratory) and Malaria (the Malaria Office); and (ii) Ovicol, AGO and BG-Sentinel (BGS) traps were tested in hotels, schools, churches, pharmacies and government buildings (Fig. 2).

In Santa Marta, 500 Ovicol traps were deployed. In San José del Guaviare, 60 Ovicol traps with oviposition substrates (white paper napkins and white nonwoven sheets) and the following bioattractants were prepared:

(i) Bird seed (*Ph. canariensis*)—whole grains (1 g) or fermented extract (100 g/5 l water, 8-day incubation; 5 ml per trap)
(ii) Panela (grafted unrefined cane sugar): 1 g with 0.2 g dry yeast (*Saccharomyces cerevisiae*)
(iii) Blackstrap (*Saccharum officinarum*)—diluted 1:10 in water and enriched with dry yeast (*Sa. cerevisiae*, 0.2 g; 5 ml of the diluted molasses per trap).

Larvicides were supplied by the ETS: diflubenzuron–Dimilin® (a chitin synthesis inhibitor) and *Bacillus thuringiensis israelensis* (BTi, granular formulation), both of which were dosed according to WHO guidelines [24]. All Ovicol traps were filled with 250 ml of untreated tap water and labeled alphanumerically with colored tags for traceability.

### Procedures by site

In Santa Marta, 100 Ovicol traps were installed at each of the five site: 50 traps containing molasses + yeast (M + Y) as bioattractant and 50 traps containing bird seed (BS) as bioattractant; all traps were treated with diflubenzuron. Weekly monitoring was performed by teams of one professional and two environmental technicians. The number of eggs per trap was counted in situ after removal of the oviposition substrate. The paper oviposition substrate in each trap was replaced at each visit, while attractants and larvicides were renewed biweekly. At the end of each sampling event, egg subsamples were taken to the laboratory for testing, and the remaining paper/oviposition substrates were inactivated with hot water or boiled for 10 min. In a previous study, we demonstrated that hot water inactivates 100% of eggs [21].

In San José del Guaviare, a total of 60 of 500 Ovicol units were deployed, of which 30 were assigned to the institutional evaluation component (Public Health Laboratory and Malaria Office), and 30 were used in the comparative evaluation with AGO and BGS traps. In addition, 30 AGO and 30 BGS traps were installed. All traps were inspected at 24, 48 and 72 h and 7 and 15 days post-installation by two teams of three members each. The collected samples were transported to the Departmental Medical Entomology Laboratory for egg counting and taxonomic identification. The AGO and BGS traps used the same attractants but no larvicides since AGO relies on adhesive surfaces and BGS on electrical suction. A water-only control was excluded because it was found in a previous study to have low oviposition positivity [21].

Taxonomic identification of larvae (post-hatching) and adults was performed via standard taxonomic keys [25, 26]. After each monitoring round, all oviposition substrates were boiled for 5 min to ensure final inactivation of residual eggs.

### Variables and statistical analyses

The dependent variables included the number of eggs, immature stages, adult mosquitoes and species composition. The independent variables included trap type (Ovicol/AGO/BGS) and treatment (bird seed/fermented bird seed/molasses + yeast/water).

The egg-related outcomes were quantified using the following indices: (i) Oviposition Positivity Index (OPI), which indicates the percentage of traps positive for *Aedes* eggs per total number of functional traps inspected per monitoring period; and (ii) egg density per trap (IDH), which is the mean number of *Aedes* eggs per positive trap

Descriptive and inferential analyses were performed, including analysis of variance (ANOVA) or nonparametric tests (Kruskal–Wallis with Dunn–Bonferroni post hoc), chi-square (*χ*^2^)/Fisher tests for proportions, odds ratios (ORs), and effect sizes (*r*). Spatial logistic regression models, Moran’s *I* autocorrelation, the LISA package, Getis-Ord Gi* spatial statistics tool and geospatial clustering analyses were applied via Python 3.10 [27].

## Results

### Santa Marta: programmatic performance of Ovicol traps

A total of 41,677 eggs were inactivated over a 5-week period. Taxonomic confirmation of larval subsamples (post-hatching) verified that 100% of the samples belonged to *Ae. aegypti*.

**Fig. 3:**
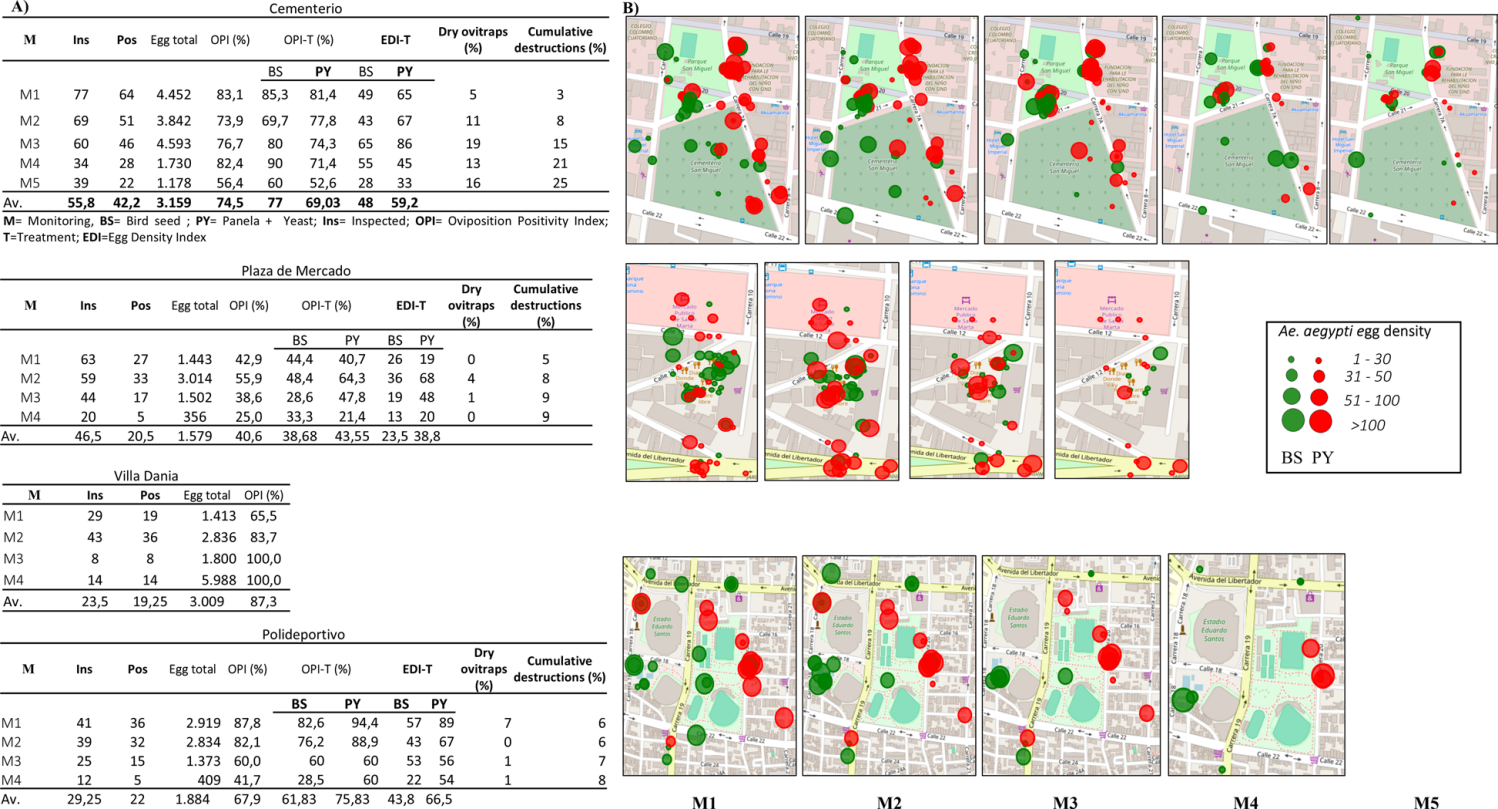
Entomological performance indicators of Ovicol traps with two bioattractants (bird seed and panela + yeast) (**a**) and spatial distribution of *Aedes aegypti* egg density in Ovicol traps placed in public areas/civic spaces of Santa Marta (**b**). Cementerio, San Miguel Cemetery; Plaza de Mercado, central marketplace; Villa Dania, peridomestic area of Villa Dania neighborhood; Polideportivo, municipal sports complex

All scheduled monitoring/collection activities were completed at three of the five (60%) sites (San Miguel Cemetery, central marketplace and municipal sports complex), while the scheduled monitoring/collection activities were only partially performed at one site (Villa Dania), and compliance failed at the Water Park (Parque del Agua) site. Among the three fully monitored sites, the OPI ranged from 40.6% to 74.5%. Trap loss due to destruction or removal was recorded at all sites, reaching 88% at the municipal sports complex (Fig. 3).

Both attractants were effective, although a consistent trend toward higher egg density per trap (IDH) was observed in those containing panela + yeast, with statistically significant differences only in traps at the municipal sports complex (Polideportivo) site (Fig. 1d). No pupae were reported throughout the monitoring period, indicating that larvicidal treatment effectively prevented adult emergence.

### San José del Guaviare

#### Ovicol and biottractants

**Fig. 4:**
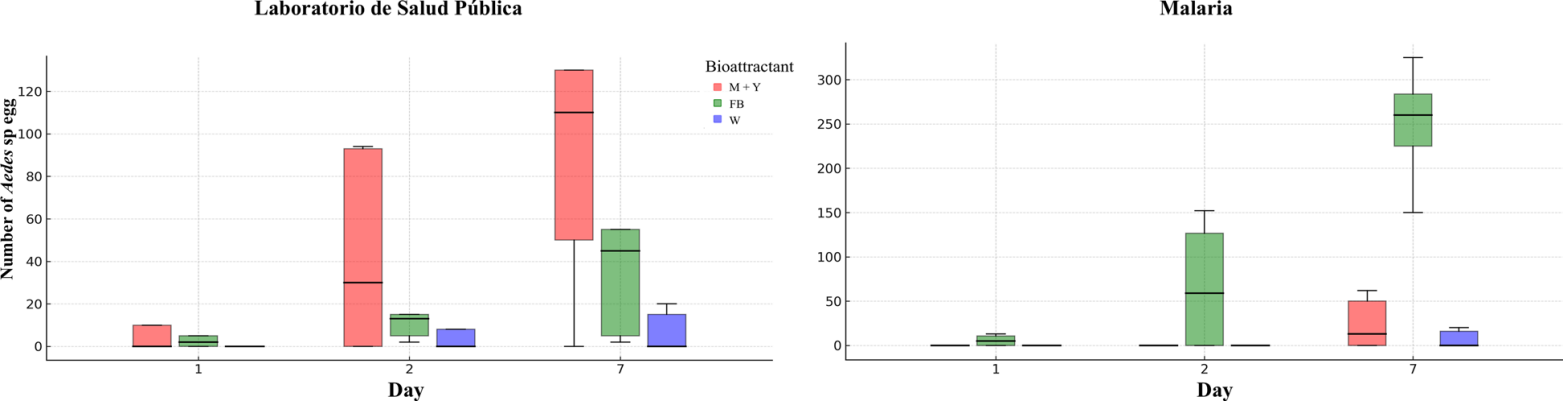
Boxplot of temporal dynamics of *Aedes* spp. oviposition in Ovicol traps using two types of bioattractants in public establishments of San José del Guaviare, Colombia. FB, Fermented bird seed bioattractant; Laboratorio de Salud Pública, Public Health Laboratory; M + Y, molasses + yeast bioattractant; Malaria, the Malaria Office; W, water

*Aedes* spp. oviposition was detected in Ovicol traps containing fermented bird seed (FB) and M + Y, respectively, within 24 h of installation. By day 7 post-placement, significantly higher OPIs were recorded for traps with the bioattractants compared to those containing only water (Kruskal–Wallis, *P* < 0.05). Similar patterns were observed for the IDH. At the Public Health Laboratory site, IDH values showed partial overlap among bioattractants, with an average of 112 eggs per positive trap (95% confidence interval [CI] 45–230). In contrast, at the Malaria Office site, markedly higher egg densities were obtained with the FB bioattractant, with a mean of 249 eggs per positive trap (95% CI 170–360); these confidence intervals were non-overlapping relative to the other attractants (Fig. 4).

.

In addition, one *Ae. albopictus* female and one *Culex* (*Melanoconion*) sp. female were captured with a handheld aspirator above the M + Y and water (W) substrates, respectively. By day 7, 73.3% (11/15) of the Ovicol traps at the Public Health Laboratory site had completely dried out, a situation not observed at the Malaria Office site.

#### Ovicol traps versus AGO traps

Between 80% and 93% of the traps were successfully inspected on day 7. In the AGO traps, the addition of bioattractants increased *Aedes* spp. detection by 91.7% relative to the control traps containing only water. The FB attractant (83.3%; 95% CI 51.6–97.9%) was 48% more effective than the M + Y attractant (35.7%; 95% CI: 12.8–64.9%) in detecting *Aedes* mosquitoes, whereas water performed 2.7-fold better than both bioattractants in terms of *Culex* spp. detection (Kruskal–Wallis *H* = 9.42; *P* = 0.009).

Post hoc analysis with Dunn–Bonferroni correction confirmed that the FB bioattractant was significantly more positive than both water (*P* = 0.022) and M + Y (*P* = 0.035), whereas no significant differences were found between M + Y and water (*P* = 0.84) (Table 1).

**Table 1:** Comparative performance of Ovicol traps and autocidal gravid ovitraps for the surveillance and control of synanthropic *Aedes* and *Culex* mosquitoes in San José del Guaviare, 2025

Trap type	Bioattractant	Number of traps Installed	Number of traps inspected (%)	Total individuals (*n*)^a^		% OPI^b^	Capture rate^c^	Statistical significance^d^
				*Aedes* sp.	*Culex* sp.			
AGO	W	10	9 (90)	3	6	11.11 (1/9)	NA	X
	FB	15	12 (80)	14	6	25.0 (3/12)	3.5	X
	M + Y	15	12 (80)	9	3	33.3 (4/12)	2.25	X
OVICOL	FB	15	12 (80)	909	0	83.33 (10/12)	90.9	*P* < 0.001
	M + Y	15	14 (93.3)	394	0	35.7 (5/14)	79	

*AGO* Autocidal gravid ovitrap,* FB* fermented seed,* M + Y* molasses + yeast,* NA* data not available,* OPI* Oviposition Positivity Index

^a^Adults were collected in the AGO trap and eggs were collected in the Ovicol trap

^b^Positivity of adults in AGO and eggs in Ovicol for *Aedes* spp

^c^Mean number of individuals per positive ovitrap (*Aedes* only)

^d^Values followed by same lowercase letter within column are not significantly different (*P* < 0.05)

^e^Matching letters vertically indicates no significant differences

Overall, Ovicol traps (OPI: 65.2%) were 2.1-fold more effective than AGO traps (OPI: 30.8%) in detecting *Aedes* mosquitoes (*P* < 0.001, *r* = 0.87; 95% CI 1.02–10.85). Within Ovicol traps, FB induced higher oviposition activity than M + Y, as reflected by both a higher OPI and egg density per positive trap (IDH: 90.9 eggs/trap, 95% CI 63.2–129.7 vs. 78.8, 95% CI 49.8–122.6) (Table 2).

**Table 2:** Co-positivity for *Aedes* spp. according to the distance between paired Ovicol and autocidal gravid ovitraps traps

Distance	Paired Ovicol/AGO traps (*n*)	% Ovicol	% AGO	% Co-positivity
< 5 m	10	70.0 (7/10)	40.0 (4/10)	40.0 (4/10)
5–30 m	7	85.1 (6/7)	57.1 (4/7)	57.1 (4/7)
> 30 m	11	54.5 (6/11)	27.3 (3/11)	9.1 (1/11)

*AGO* Autocidal gravid ovitrap

The superiority of Ovicol traps was spatially consistent, both when it was installed at the same location as the AGO traps and when it was positioned more than 30 m apart. Co-positivity between both trap types was 6.3-fold greater at distances < 30 m (57.1%) than at distances > 30 m (9.1%) (*χ*^2^ = 5.92; *P* = 0.052; Cramer’s *V* = 0.35). Spatial interference tests revealed no negative effect of AGO traps on Ovicol performance (OR 1.67; *P* = 0.68, Fisher’s exact test), indicating that AGO trap positivity did not reduce Ovicol positivity.

Ovicol traps detected a microfocus of infestation (egg clustering) (Moran’s *I* = 0.38; *P* = 0.002) that spatially coincided with the highest AGO positivity zone; notably, whenever an AGO trap captured adult mosquitoes, the corresponding Ovicol was 100% positive.

Spatial logistic regression further revealed that the FB bioattractant increased the likelihood of Ovicol positivity by 18.9-fold (OR 18.9; *P* = 0.003). Getis-Ord Gi* analysis revealed a significant oviposition hotspot at the Guaviare River port or landing area (*P* = 0.03), where all Ovicol traps (100%) were positive.

#### Ovicol versus BGS traps: both using M + Y attractant

Among the 30 BGS traps, 10% were disconnected from power after 24 h and 19.4% after 48 h; by the end of the weekend (day 5), > 70% of the traps had been turned off—mainly because of increased electrical consumption and the closure of premises during nonworking hours. Despite Ovicol and BGS traps being located in the same premises, Ovicol trap monitoring was substantially more efficient (96.6%) since they were installed outdoors on terraces, whereas BGS units were placed indoors for security reasons.

Within 48 h, 12 mosquito morphospecies (Culicidae) were recorded, of which seven were shared over both trap types and five were exclusive to BGS traps. The most abundant species were *Culex quinquefasciatus*, *Ae. aegypti* and *Ae. albopictus*, and the capture rates of these three mosquito species were approximately 30-fold greater in BGS traps than in Ovicol traps. *Psorophora ferox* was detected only in Ovicol traps, whereas *Toxorhynchites hemorrhoidalis* was unique to BGS traps (Table 3).

**Table 3:** Total abundance of *Culicidae* species by trap type (BG-Sentinel vs. Ovicol)

Mosquito species	BG-Sentinel (BGS)			Ovicol			Ratio (BGS/Ovicol)
	Total^a^	Female	Male	Total	Female	Male	
*Culex quinquefasciatus*	156	35	38	2	1	1	78.0
*Aedes aegypti*	95	54	23	3	1	2	31.7
*Aedes albopictus*	65	22	10	2	2	0	32.5
*Culex* spp.	61	–	–	10	1	3	6.1
*Culex melanoconion* sp.	50	2	–	9	3	1	5.6
*Aedes* spp.	6	–	–	0	0	0	NA
*Culex melanoconion ocossa*	4	4	0	0	0	0	NA
*Toxorrhynchites hemorrhoidalis*	2	–	–	0	0	0	NA
*Psorophora janthinesoma albigenum*	1	1	0	0	0	0	NA
*Psorophora albipes*	1	1	0	0	–	–	NA
*Psorophora ferox*	1	1	0	2	2	0	0.5
*Psorophora* sp.	1	–	–	1	–	–	1.0
*Mansonia* sp.	1	–	–	0	0	0	

Values in table are frequency (*n*)

*BGS* BG-Sentinel,* NA* Data not available

^a^It was not possible to determine the sex of all individuals owing to the absence of the morphological structures required for accurate differentiation

The Ovicol trap had an OPI of 20.0% (6/30) at 24 h (97 *Aedes* eggs) and 35.5% (11/31) at 48 h (223 cumulative eggs). Compared with the BGS trap as the reference standard, Ovicol had a sensitivity of 25%, specificity of 57% and overall concordance of 46%. The Cohen’s kappa coefficient (− 0.17) indicated poor agreement, even below that expected by chance. Spatial statistics corroborated this lack of association: there was no spatial correlation between BGS adult captures and Ovicol oviposition (slope: − 0.59; *R*^2^ 0.02). The Moran’s *I* values were − 0.08 for adult *Aedes* (*P* = 0.80) and − 0.01 for eggs (*P* = 0.26), with no significant local clusters (LISA, *P* > 0.10).

## Discussion

The field evaluation of Ovicol traps conducted under the operational conditions of Colombia’s national vector surveillance and control programs provides several relevant insights when compared with AGO and BGS traps. Across two territorial health entities (ETSs), Ovicol demonstrated high operational feasibility and rapid responsiveness. In Guaviare, *Aedes* oviposition was detected within the first 24 h, whereas in Santa Marta, over a 5-week period, the intervention enabled the removal and thermal inactivation (with hot water) of 43,203 *Ae. aegypti* eggs. On the basis of the previously established entomological indicator—i.e. for every 100 *A. aegypti* eggs removed, the emergence of 71 adults is prevented [21]—the intervention likely prevented the emergence of more than 30,000 adults in just 5 weeks.

Both the FB and M + Y (molasses + yeast) bioattractants proved to be cost-effective, logistically simple, biodegradable and extremely low-cost (< USD 0.01 per trap). This contrasts with BGS traps, which use industrial attractants that cost approximately US$ 28 per unit (https://research-shop.biogents.com/collections/mosquito-attractants). This affordability makes Ovicol particularly scalable in resource-limited settings across more than 140 dengue-endemic countries worldwide [28].

Our observations are highly consistent with the results of studies conducted in China comparing improved versions of handmade ovitraps (OT), such as the Mosq-ovitrap (MOT), with industrial devices, such as CO₂-light traps (CLT) [29], which showed that oviposition-based traps were more sensitive and cost-effective for mosquito detection than industrial traps. In particular, the optimized MOT achieved egg density values of 12.38 eggs per trap after 3 days of exposure and 34.15 eggs per trap after 7 days. Taken together, these results and those of the present study demonstrate that ovitraps and their variants, such as Ovicol, are sensitive within the first 24 h and represent a cost-effective surveillance tool across different regions worldwide.

In summary, Ovicol combines ease of deployment, early sensitivity and minimal marginal costs and is, consequently, a desirable combination for *Aedes* surveillance and control under programmatic conditions.

In a direct comparison, Ovicol traps had higher OPI for *Aedes* than did AGO traps in Guaviare (65.2% vs. 30.8%), with no evidence of trap interference. Co-positivity decreased with distance, and the Ovicol-detected oviposition hotspot spatially coincided with the area with the highest AGO positivity, suggesting functional complementarity: AGO captures gravid females (adults), whereas Ovicol indices oviposition (eggs). This pattern is consistent with results from comparative studies conducted in Brazil that evaluated larval *Aedes* indices (presence–absence), conventional OT and sticky OT (MosquiTRAP), which demonstrated that OT were more effective for *Aedes* vector surveillance, showing up to 50% higher sensitivity than MosquiTRAP [30].

The combined use of oviposition and adult-based indices enhances spatiotemporal resolution for the early detection of microfoci of infestation and supports the targeted implementation of control actions. Moreover, Ovicol promotes the rational use of larvicides (diflubenzuron/BTi) by functioning as a lethal trap that competes with cryptic or residential breeding sites. The conventional approach applies one diflubenzuron tablet per 200 l of water [24], which is enough to treat approximately one household. In contrast, each Ovicol trap uses only 0.25 l of water, allowing a single tablet to treat approximately 800 Ovicol traps, achieving an equivalent coverage of approximately 750–800 households. This represents an approximately 800-fold increase in treatment efficiency and spatial coverage while substantially reducing the environmental chemical load and operational costs. Furthermore, Ovicol can be adapted as an autodissemination ovitrap via growth regulators such as pyriproxyfen [31], which aligns with the new IVM policy framework [15].

Although BGS traps capture a greater abundance and diversity of adult mosquitoes, which is consistent with their design for adult surveillance [14, 32], they are associated with operational limitations. Electrical disconnections (≥ 70% by the end of the week) compromised study continuity in key establishments (hotels, restaurants, healthcare institutions, etc.). These observations are consistent with reports from the USA where BGS traps captured a higher diversity and abundance of adult mosquitoes, while oviposition cups showed limited ability to reflect differences in adult population structure between *Ae. aegypti* and *Ae. albopictus* [33]. In our study, however, Ovicol not only demonstrated high sensitivity for oviposition detection but also captured resting adults of multiple mosquito species, including *Ae. aegypti*, *Ae. albopictus*, *Cx.* (*Melanoconion*) sp., and *Ps. ferox*. While Ovicol traps are not intended to replace BGS traps for detailed entomological characterization, these observations indicate that oviposition-based traps may provide complementary information on both reproductive activity and adult presence under operational field conditions.

The early detection (24–48 h) of oviposition activity by *Ae. aegypti* and *Ae. albopictus* underscores the potential of the Ovicol trap as a valuable tool during recent yellow fever outbreaks in South America [23] and amid the largest recorded dengue epidemic in the Americas, with more than 13 million cases and 8431 associated deaths [34]. Given the growing evidence of transovarial transmission of dengue and yellow fever viruses by *Aedes*—with both classical and recent reports confirming infected eggs from infected females [35–37]—our findings support the integration of the Ovicol trap into entomovirus surveillance programs, including arbovirus tracking, estimation of natural infection rates and prediction of human transmission hotspots. Thus, the Ovicol trap provides a complementary risk indicator to adult mosquito capture, with a significant logistical advantage for high-volume sampling.

Several programmatic challenges were identified during implementation. Trap losses due to vandalism or removal—reaching up to 88% of Ovicol traps at one Santa Marta site—highlight the need for community engagement, trap labeling and neighborhood participation mechanisms. The literature consistently shows that sustained community participation can reduce vector density and even outperform chemical interventions [38, 39]. Additionally, there were gaps in program management in Santa Marta, with only 60% of activities completed by the monitoring team, and no team exceeded 5 weeks of monitoring. This suggests the need for stronger ministerial leadership, with clear entomological targets, process/outcome indicators and medium- to long-term monitoring cycles. These findings mirror international reports showing that over 97% of *Aedes* control actions in endemic countries lack quality indicators or pre/post-intervention evaluations [38, 39].

Finally, this study has limitations inherent to real-world operational settings, including trap losses, and microenvironmental heterogeneity (shade, sunlight exposure, rainfall). In Santa Marta, no water-only control was included—justified by previously documented low egg positivity—although this limits absolute comparisons. Taxonomic identification was performed on subsamples (larvae post-hatching and adults). Future studies should incorporate longer time series, climatic covariates and quasi-experimental designs with microhabitat blocking, as well as automated egg counting systems, with the aim to reduce human bias and improve precision in large-scale monitoring.

## Data Availability

Data supporting the main conclusions of this study are included in the manuscript.

## References

[1] Walter Reed Biosystematics Unit. *Aedes* genus page. Walter Reed Biosystematics Unit Website; 2020. https://wrbu.si.edu/vectorspecies/genera/aedes. Accessed 23 Apr 2025.

[2] Huang Y-JS, Higgs S, Vanlandingham DL. Arbovirus-mosquito vector-host interactions and the impact on transmission and disease pathogenesis of arboviruses. Front Microbiol. 2019;10:22. 10.3389/fmicb.2019.00022. 30728812 10.3389/fmicb.2019.00022 PMC6351451

[3] Powell JR, Gloria-Soria A, Kotsakiozi P. Recent history of *Aedes aegypti*: vector genomics and epidemiology records. Bioscience. 2018;68:854–60. 10.1093/BIOSCI/BIY119. 30464351 10.1093/biosci/biy119 PMC6238964

[4] Pichler V, Kotsakiozi P, Caputo B, Serini P, Caccone A, Torre A Della. Complex interplay of evolutionary forces shaping population genomic structure of invasive Aedes albopictus in southern Europe. PLoS Negl Trop Dis. 2019;13:e0007554. 10.1371/JOURNAL.PNTD.0007554. 31437154 10.1371/journal.pntd.0007554 PMC6705758

[5] Wilkerson RC, Linton Y-M, Strickman D. Mosquitoes of the world. In: Mosquitoes of the world. Baltimore: Johns Hopkins University Press; 2021. 10.1353/BOOK.79680.

[6] Roiz D, Pontifes PA, Jourdain F, Diagne C, Leroy B, Vaissière AC, et al. The rising global economic costs of invasive *Aedes* mosquitoes and *Aedes*-borne diseases. Sci Total Environ. 2024;933:173054. 10.1016/j.scitotenv.2024.173054. 38729373 10.1016/j.scitotenv.2024.173054

[7] Sparks TC, Nauen R. IRAC: mode of action classification and insecticide resistance management. Pestic Biochem Physiol. 2015;121:122–8. 10.1016/j.pestbp.2014.11.014. 26047120 10.1016/j.pestbp.2014.11.014

[8] PAHO, WHO. Epidemiological alert Yellow fever in the Americas Region. Washington, D.C; 2025.

[9] PAHO. Epidemiological alert Yellow fever in the Americas Region. 3 February 2025.

[10] Damasceno-Caldeira R, Nunes-Neto JP, Aragão CF, Freitas MNO, Ferreira MS, Castro PHG de, et al. Vector competence of *Aedes albopictus* for Yellow Fever virus: risk of reemergence of urban Yellow Fever in Brazil. Viruses. 2023;15:1019. 10.3390/V15041019. 37112999 10.3390/v15041019 PMC10146658

[11] Soper FL. The elimination of urban Yellow Fever in the Americas through the eradication of *Aedes aegypti*. Am J Public Health Nation’s Health 1963;53:7–16. 13978257 10.2105/ajph.53.1.7 PMC1253857

[12] Montenegro D, Martinez L, Tay K, Hernandez T, Noriega D, Barbosa L, et al. Usefulness of autocidal gravid ovitraps for the surveillance and control of *Aedes* (*Stegomyia*) *aegypti* (Diptera: Culicidae) in eastern Colombia. Med Vet Entomol. 2020;34:379–84. 10.1111/mve.12443. 32232987 10.1111/mve.12443

[13] Barrera R, Acevedo V, Felix GE, Hemme RR, Vazquez J, Munoz JL, et al. Impact of autocidal gravid ovitraps on Chikungunya virus incidence in *Aedes aegypti* (Diptera: Culicidae) in areas with and without traps. J Med Entomol. 2017;54:387–95. 10.1093/jme/tjw187. 28031347 10.1093/jme/tjw187 PMC6505457

[14] Maciel-de-Freitas R, Eiras ÁE, Lourenço-de-Oliveira R. Field evaluation of effectiveness of the BG-Sentinel, a new trap for capturing adult *Aedes aegypti* (Diptera: Culicidae). Mem Inst Oswaldo Cruz. 2006;101:321–5. 10.1590/S0074-02762006000300017. 16862330 10.1590/s0074-02762006000300017

[15] OPS. Evaluación de las estrategias innovadoras para el control de Aedes aegypti: desafíos para su introducción y evaluación del impacto. Washington: Orgización Panamericana de Salud; 2019.

[16] OPS. Documento operativo de aplicación del manejo integrado de vectores adaptado al contexto de las Américas. Washington D.C.: Organización Panamericana de la Salud.; 2019.

[17] OPS. Documento técnico para la implementación de intervenciones basado en escenarios operativos genéricos para el control del Aedes aegypti. vol. 1. 1st ed. Washington; 2019.

[18] Navarro-Kraul JI, Vázquez LAC, Paiz-Moscoso KE, Danis-Lozano R, Dávila-Barboza JA, Lopez-Monroy B, et al. The field assessment of quiescent egg populations of *Aedes aegypti* and *Aedes albopictus* during the dry season in Tapachula, Chiapas, Mexico, and its potential impact on vector control strategies. Insects. 2024;15:798. 10.3390/INSECTS15100798. 39452374 10.3390/insects15100798 PMC11508872

[19] Reiter P, Sprenger D. The used tire trade: a mechanism for the worldwide dispersal of container breeding mosquitoes. J Am Mosq Control Assoc. 1987;3:494–501. 2904963

[20] Finlay C. El mosquito hipoteticamente considerado como agente de transmisión de la fiebre amarilla. Salud Publica Mex. 1992;34:474–83. 1354395

[21] Arrieta-Ángel K, Polo-Silva K, Ospino-Sierra N, Corpas-Choperena D, Monterrosa E, Alemán I, et al. Ovitrampas artesanales y agua caliente: estrategia auxiliar para el control del mosquito *Aedes aegypti* en Colombia. Rev Panam Salud Publica. 2025;49:1–10. 10.26633/RPSP.2025.40. 10.26633/RPSP.2025.40 PMC12051641 40330805

[22] INS. Dengue: situación epidemiológica en Colombia. Instituto Nacional de Salud; 2024. https://app.powerbi.com/view?r=eyJrIjoiOTIxMzE4MGItNjg4MC00ZmUyLWIwMzctODhlOWFjNzMyZmViIiwidCI6ImE2MmQ2YzdiLTlmNTktNDQ2OS05MzU5LTM1MzcxNDc1OTRiYiIsImMiOjR9. Accessed 29 May 2024.

[23] OPS/OMS. Actualización Epidemiológica. Fiebre amarilla en la Región de las Américas, 24 de abril del 2025. Washington, D.C; 2025.

[24] WHO. Report of the ninth WHOPES working group meeting: WHO/HQ, Geneva, 5–9 December 2005: review of: Dimilin GR and DT, Vectobac DT, Aqua K-Othrine, Aqua Reslin Super. Geneva; 2006.

[25] En A, Gustavo U, Rossi C, Martínez M. Lista de especies y clave ilustrada para la identificación de larvas de mosquitos (Diptera: Culicidae) halladas criando en recipientes artificiales en Uruguay. Bol Soc Zool. 2016;22:49–65.

[26] Villarroel E. Taxonomía y bionomía de los géneros de Culicidae (diptera: nematocera) de Venezuela: desarrollo de una clave fotográfica. Universidad Central de Venezuela; 2013.

[27] Van Rossum G, Drake Jr FL. Python reference manual. PythonOrg. 1995. https://www.python.org/. Accessed 2 May 2025.

[28] OMS. Dengue y dengue grave. Organización Mundial de La Salud 2019. https://www.who.int/es/news-room/fact-sheets/detail/dengue-and-severe-dengue. Accessed 4 Aug 2019.

[29] Gao Q, Cao H, Fan J, Zhang Z, Jin S, Su F, et al. Field evaluation of Mosq-ovitrap, Ovitrap and a CO2-light trap for Aedes albopictus sampling in Shanghai, China. PeerJ. 2019;2019:e8031. 10.7717/PEERJ.8031/SUPP-3. 10.7717/peerj.8031 PMC6884993 31799071

[30] de Resende MC, Silva IM, Ellis BR, Eiras ÁE. A comparison of larval, ovitrap and MosquiTRAP surveillance for Aedes (Stegomyia) aegypti. Mem Inst Oswaldo Cruz 2013;108:1024. 10.1590/0074-0276130128. 10.1590/0074-0276130128 PMC4005541 24402144

[31] Devine GJ, Perea EZ, Killeen GF, Stancil JD, Clark SJ, Morrison AC. Using adult mosquitoes to transfer insecticides to *Aedes aegypti* larval habitats. Proc Natl Acad Sci USA. 2009;106:11530–4. 10.1073/PNAS.0901369106. 19561295 10.1073/pnas.0901369106 PMC2702255

[32] Farajollahi A, Kesavaraju B, Price DC, Williams GM, Healy SP, Gaugler R, et al. Field efficacy of BG-Sentinel and industry-standard traps for *Aedes albopictus* (Diptera: Culicidae) and West Nile Virus surveillance. J Med Entomol. 2009;46:919–25. 10.1603/033.046.0426. 19645298 10.1603/033.046.0426

[33] Wright JA, Larson RT, Richardson AG, Cote NM, Stoops CA, Clark M, et al. Comparison of BG-Sentinel® trap and oviposition cups for *Aedes aegypti* and *Aedes albopictus* surveillance in Jacksonville, Florida, USA. J Am Mosq Control Assoc. 2015;31:26–31. 10.2987/14-6434R.1. 25843173 10.2987/14-6434R.1

[34] PAHO. PLISA health information platform for the Americas. PAHO/WHO 2025;1. https://www.paho.org/data/index.php/en/mnu-topics/indicadores-dengue-en/dengue-nacional-en/252-dengue-pais-ano-en.html. Accessed 23 July 2019.

[35] Janjoter S, Kataria D, Yadav M, Dahiya N, Sehrawat N. Transovarial transmission of mosquito-borne viruses: a systematic review. Front Cell Infect Microbiol. 2023;13:1304938. 10.3389/FCIMB.2023.1304938/BIBTEX. 38235494 10.3389/fcimb.2023.1304938 PMC10791847

[36] Cruz ACR, Hernández LHA, Aragão CF, da Paz TYB, da Silva SP, da Silva FS, et al. The importance of entomo-virological investigation of yellow fever virus to strengthen surveillance in Brazil. Trop Med Infect Dis. 2023;8:329. 10.3390/TROPICALMED8060329/S1. 37368747 10.3390/tropicalmed8060329 PMC10305592

[37] Aitken THG, Tesh RB, Beaty BJ, Rosen L. Transovarial transmission of yellow fever virus by mosquitoes (*Aedes aegypti*). Am J Trop Med Hyg. 1979;28:119–21. 10.4269/AJTMH.1979.28.119. 434305 10.4269/ajtmh.1979.28.119

[38] Bouzid M, Brainard J, Hooper L, Hunter PR. Public health interventions for *Aedes* control in the time of Zikavirus—a meta-review on effectiveness of vector control strategies. PLoS Negl Trop Dis. 2016;10:e0005176. 10.1371/JOURNAL.PNTD.0005176. 27926934 10.1371/journal.pntd.0005176 PMC5142773

[39] Alvarado-Castro V, Paredes-Solís S, Nava-Aguilera E, Morales-Pérez A, Alarcón-Morales L, Balderas-Vargas NA, et al. Assessing the effects of interventions for *Aedes aegypti* control: systematic review and meta-analysis of cluster randomised controlled trials. BMC Public Health. 2017;17:384. 10.1186/s12889-017-4290-z. 28699552 10.1186/s12889-017-4290-z PMC5506587

